# Establishment of the Primary Avian Gonadal Somatic Cell Lines for Cytogenetic Studies

**DOI:** 10.3390/ani12131724

**Published:** 2022-07-04

**Authors:** Inna E. Pristyazhnyuk, Lyubov P. Malinovskaya, Pavel M. Borodin

**Affiliations:** 1 Department of Molecular Genetics, Cell Biology and Bioinformatics, Institute of Cytology and Genetics, Siberian Department of Russian Academy of Sciences, 630090 Novosibirsk, Russia; iprist@bionet.nsc.ru; 2 Department of Cytology and Genetics, Faculty of Natural Sciences, Novosibirsk State University, 630090 Novosibirsk, Russia; l.malinovskaia@g.nsu.ru

**Keywords:** avian primary cell culture, songbirds, karyotyping, electroporation

## Abstract

**Simple Summary:**

We developed a simple method for primary somatic cell culture establishment from the ovaries of the great tits and testes of ten Passerine species. The ovary-derived cell cultures were cultivated until the tenth passage without any noticeable decrease in their proliferative activity, while testis-derived cell cultures demonstrated a decreased proliferation potential. However, sufficient material was available from both cell cultures originating from the ovary and testis to make excellent mitotic metaphase chromosomal preparations. We demonstrated the high efficiency of electroporation for genetic modification of the ovary-derived cell line. Thus, the established ovary-derived cell line could be efficiently used in cytogenetic and genomic studies.

**Abstract:**

The last decade was marked by a steep rise in avian studies at genomic and cellular levels. Cell lines are important tools for in vitro studies in cell biology and cytogenetics. We developed a simple method of primary somatic cell culture establishment from the ovaries of the great tits (*Parus major*) and testes of ten Passerine species, characterized the cellular composition of the ovary-derived lines using RT-PCR and immunolocalization of the tissue-specific markers and tested the efficiency of two methods of genetic transformation of the ovary-derived cell line. We found that the ovary-derived cell cultures of the great tit were composed of fibroblasts mainly, but also contained interstitial and granulosa cells. They were cultivated until the 10th passage without any noticeable decrease in their proliferative activity. The testis-derived cell cultures had lower proliferative potential. However, both ovary- and testis-derived cell cultures provided enough material for high quality mitotic metaphase chromosome preparations. The efficiency of its transduction with lentivirus containing a GFP reporter was very low, while electroporation with episomal vectors expressing GFP resulted in a high yield of GFP-positive cells. The proposed method could be used for the generation of high quality material for various cytogenetic and genomic studies.

## 1. Introduction

The last decade was marked by a steep rise in avian studies at the genomic, chromosomal, cellular and organismal levels [1,2,3,4]. Yet, few avian cell lines have been established. Most of them are derived from domestic (chicken, duck, Japanese quail, turkey) and model (zebra finch, canary) species [5,6,7,8,9]. The usual sources of the avian cell lines are skin, feather pulp, or blood [10,11]. However, the establishment of such cultures is a long and difficult process. More efficient and fast growing cell cultures can be established from bird embryos at the early developmental stage [11,12,13]. However, this material is difficult to obtain. Gonads also contain somatic cells with a high proliferation rate. Stable gonadal somatic cell lines from chickens, swans, geese and Japanese quail have been established [14,15,16,17,18,19].

The aim of this study was to develop a simple and efficient method for the establishment and maintenance of avian gonadal somatic cell lines, estimate the cellular composition and growth rate of the resulting lines and their utility for cytogenetic and gene engineering studies.

## 2. Materials and Methods

### 2.1. Specimens

In this study, we used four adult female great tits and one adult male of each of the following species: great tit, star finch, zebra finch, Gouldian finch, common chaffinch, brambling, European greenfinch, domestic canary, European goldfinch and common linnet. The sources of the material are shown in Table S1. The birds were handled and euthanized in accordance with the approved national guidelines for the care and use of laboratory animals. The euthanization was performed by isoflurane overdose. The study was reported in accordance with ARRIVE guidelines [20]. Ethics approval was granted by the Animal Care and Use Committee of the Institute of Cytology and Genetics SB RAS (protocol # 114 of 17 December 2021).

### 2.2. Primary Cell Culture Establishment

The gonads were isolated immediately after euthanasia and transferred into three changes of PBS. The tissue samples were minced into small pieces and transferred to the 5-mL tube. The samples were treated with 100 ng/mL collagenase I (cat#17018-029, Gibco, Waltham, MA, USA) for 20 min and with 0.25% trypsin-EDTA solution (cat#T4549, Sigma-Aldrich, St. Louis, MO, USA) for another 20 min at 37 °C. Trypsin and collagenase solutions were inactivated by Dulbecco’s Modified Eagle’s medium (DMEM) supplemented with 10% fetal bovine serum (FBS, cat# 10437028, Gibco). The cell suspension was centrifuged at 150× *g* for 5 min. The pellet was resuspended in growth medium (DMEM supplemented with 10% FBS, 2% chicken serum (cat#16110-082, Gibco), GlutaMAX (cat# 35050038, Gibco) and 500 U/mL penicillin and streptomycin (Pen Strep, cat#15140-122, Gibco) with the addition of 50 µg/mL of antibiotic and antimycotic reagents primocin (cat#ant-pm, InVivoGen, San Diego, CA, USA) and 0.25 µg/mL amphotericin B (cat#15290-026, Gibco). The cell suspension was incubated in the 0.1% gelatin-coated well of a 6-well plate at 37 °C. The growth medium was changed every second day. When cells reached 70–80% confluence, they were passaged by treatment with 0.25% trypsin–EDTA solution at a 1:1 ratio. The next passages were performed at a 1:3 ratio every 3–4 days. Fibroblast cell cultures of the great tit and zebra finch were established from the intercostal connective tissue according to the same protocol.

### 2.3. Mitotic Metaphase Chromosome Preparation

The mitotic metaphase chromosomes were prepared from the cell cultures at the second to third passage according to the standard procedure [21]. The cells were incubated in the growth medium with 1 µg/mL ethidium bromide and 50 ng/mL colcemid (cat#10295892001, Roche, Basel, Switzerland) for 3 h and then were harvested by 0.25% Trypsin-EDTA treatment. The cells were treated with hypotonic solution (0.56% KCl) for 25 min at 37 °C and fixed in two changes of the cold freshly prepared fixative (3:1 methanol: glacial acetic acid). The cell suspension was dropped on the cold wet glass and air-dried. The slides were stained with 0.2% DAPI (4′,6-diamidino-2-phenylindole, cat#D-9542, Sigma-Aldrich, Burlington, MA, USA) solution in PBS and mounted in antifade (1% DABCO (1,4-diazabicyclo [2.2.2]octane (cat#D-2522, Sigma-Aldrich)) solution in 90% Glycerol.

### 2.4. Microscopic Image Analysis

The slides were analyzed using a Carl Zeiss Axioplan 2 microscope with a CCD camera (CV M300, JAI, Kushima, Miyazaki, Japan), CHROMA filter sets, and ISIS4 image-processing package (MetaSystems GmbH, Altlussheim, Germany).

### 2.5. Measuring the Doubling Time of the Cell Population

The cell growth rate was monitored for three days in four to eight replicates for each cell culture in the wells of xCELLigence ePlates inside the standard CO_2_ incubator. The doubling time of the cell population was calculated by the xCELLigence RTCA Software Pro (Aligent, Santa Clara, CA, USA) from the logarithmic phase of the growth curve.

### 2.6. RT-PCR Analysis of Gene Expression

Total RNA was isolated from the adult ovary and primary cell cultures using TRIzol reagent according to the manufacturer’s recommendations (cat#15596018, Thermo Fisher Scientific, Waltham, MA, USA). The cDNA was synthesized from total RNA using RevertAid RT Reverse Transcription Kit (cat#K1691, Thermo Fisher Scientific). An aliquot of 1 µL cDNA was used as a template in the subsequent PCR with primers listed in Table S2. *Actin beta* gene (*β-actin*) was used as a positive control to confirm the presence of cDNA. For each gene tested, we used reverse transcriptase minus negative control to confirm that the RT-PCR signals were not derived from the genomic DNA. The PCR products were visualized by electrophoresis on 1.5% agarose gel.

### 2.7. Immunocytochemical Analysis

The cells were cultured on the glass coverslips placed in the wells of 24-well culture plates. They were fixed in 4% paraformaldehyde for 15 min at room temperature and then rinsed in PBS. The slides were incubated in the wet chamber with primary antibodies: rabbit anti-collagen type I (1:100; Chemicon, UK) and rabbit polyclonal anti-fibronectin (1:100; Abcam, Cambridge, UK) overnight at 4 °C and with the secondary Alexa Fluor 546 goat anti-rabbit antibodies (Life technologies, cat#A-11010, Carlsbad, CA, USA) for 1 h at 37 °C. Slides were mounted with Vectashield antifade mounting medium with DAPI (Vector Laboratories, cat#H-1200, Newark, CA, USA).

### 2.8. Transduction with Lentivirus Vectors Expressing GFP

We estimated the transduction efficiency of the cell lines using a lentiviral vector with GFP according to Menzorov et al. [22] and Pristyazhnyuk and Menzorov [23] with minor modifications. The lentiviruses with GFP were produced in the Phoenix cell line using Lipofectamine 3000 reagent (cat#L3000-008, Thermo Fisher Scientific). The cells at concentration 30 × 10^3^ cells/cm^2^ were plated the day before transduction. The transduction was carried out with 10 or 50% lentivirus-containing supernatant in a growth medium without antibiotics with heat-inactivated FBS, chicken serum, and 10 μg/mL Polybrene (cat#TR-1003-G, Sigma-Aldrich). The multiplicity of infection (MOI) was estimated as previously described [23].

We used spinoculation (500 g for 45 min at 37 °C) to increase the transduction efficiency [24,25]. The multiplicity of infection (MOI) was calculated as *ln*(1 − *n*), where *n* was the fraction of GFP-positive cells estimated by fluorescence-activated cell sorting (FACS) analysis.

### 2.9. Electroporation with Episomal Vectors Expressing GFP

The electroporation was carried out at 1 × 10^5^ cells in 100 μL with 2 μg of episomal vectors expressed GFP (cat#41858, Addgene, Watertown, MA, USA) using the Neon Transfection System (Thermo Fisher Scientific) according to the manufacturer protocol. The plasmid was prepared according to the supplier’s instructions. Electroporated cells were placed into the well of a 24-well culture plate into the growth medium without antibiotics and covered with 0.1% gelatin solution. The cells after 24 h of culturing were tested for green fluorescence on a ZOE Fluorescent Cell Imager (Bio-Rad, Hercules, CA, USA). For every condition, the total cell number and the number of GFP-positive cells per field of view at the ×10 objective were counted. The number of live cells per the microscope field of view was considered as the survival rate and the percentage of GFP-positive cells as the electroporation efficiency.

## 3. Results and Discussion

### 3.1. Establishment of the Primary Cell Cultures from the Passerine Gonads

We established the four cell cultures from the ovaries of four great tits. Three of them (OFC2, OFC5 and OFC8) were established from the ovaries of adult birds captured during the breeding season (April–May 2021) and one from the ovary of a subadult female captured in September 2021 (OFC1A). We also established the ten cell cultures from the testes of the passerine species listed in Table S1.

**Figure 1:**
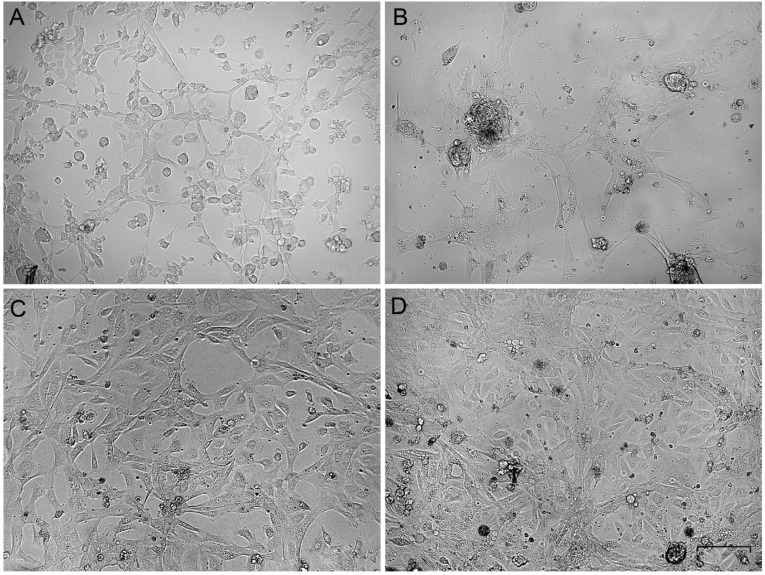
Phase-contrast micrographs of primary gonadal cell cultures on the first (**A**,**B**) and the third (**C**,**D**) passages. (**A**,**C**)—ovary-derived lines, (**B**,**D**)—testis-derived lines. Bar—100 µm.

The primary gonadal culture cells varied in morphology. Some roundish cells were present at the beginning (Figure 1A,B), but after several passages, the elongated cells gained an advantage (Figure 1C,D). The cells were subcultured at a ratio of 1:3 every third day and frozen-thawed at the standard conditions. In the third passage of all ovary-derived cell cultures, the cell morphology was stabilized as fibroblast-like and did not change during further cultivation. The cell cultures were established and became suitable for cytogenetic analysis in about 10 days.

**Figure 2:**
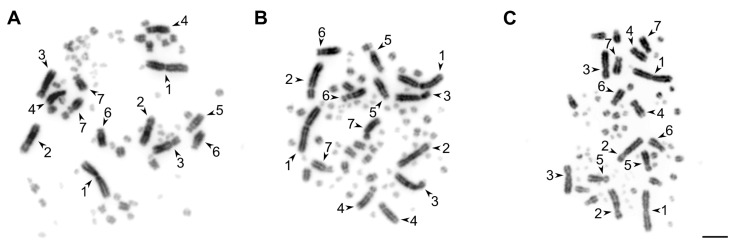
The examples of metaphase chromosome plates prepared from the short-term ovary (**A**) and testis-derived (**B**) cell cultures of the great tit and testis-derived cell culture of the starfinch (**C**). The arrowheads indicate the seven largest macrochromosomes. Bar—5 μm.

Testis-derived cell cultures have lower proliferative potential in comparison with ovary-derived ones. After the third passage, most testis-derived cell cultures lost their proliferative activity, whereas the ovary-derived cells retained it until at least the 10th passage. However, both the short-term ovary- and testis-derived cell cultures provided enough material for high quality mitotic metaphase chromosome preparations (Figure 2 and Figure S1).

Table 1 summarizes the advantages and disadvantages of various methods of bird chromosome preparation. Our method of primary cell culture establishment from the ovary and testis of the songbirds could be effectively used for the rapid generation of high quality material for various cytogenetic and genomic studies. Its main disadvantage is that it involves bird sacrifice. For this reason, it cannot be recommended for routine karyotyping during population studies and for the karyotyping of endangered species.

### 3.2. Growth Rate and Cellular Composition of the Ovary-Derived Cell Lines

The OFC1A cell line from the ovary of the subadult female captured beyond the breeding season demonstrated the highest proliferative activity among the ovary-derived cells. It did not noticeably decrease until the 10th passage of cultivation. To determine the causes of its high proliferative activity, we examined the cell composition of the OFC1A cell line and compared it with that of the OFC2 and OFC5 lines, derived from the ovaries of adult females captured during the breeding season.

The growth rate of the cell lines was estimated as the doubling time of cell populations by the xCELLigence cell analyzer. On the eighth passage, it was 32 ± 0.3, 34.6 ± 0.8 and 17.3 ± 0.3 h for OFC2, OFC5 and OFC1A lines, respectively. Thus, OFC1A cells divide twice as fast as the OFC2 and OFC5 cells (Student’s *t*-test, *p* < 0.01). Is this due to the difference in the cellular composition of the cell lines?

**Figure 3:**
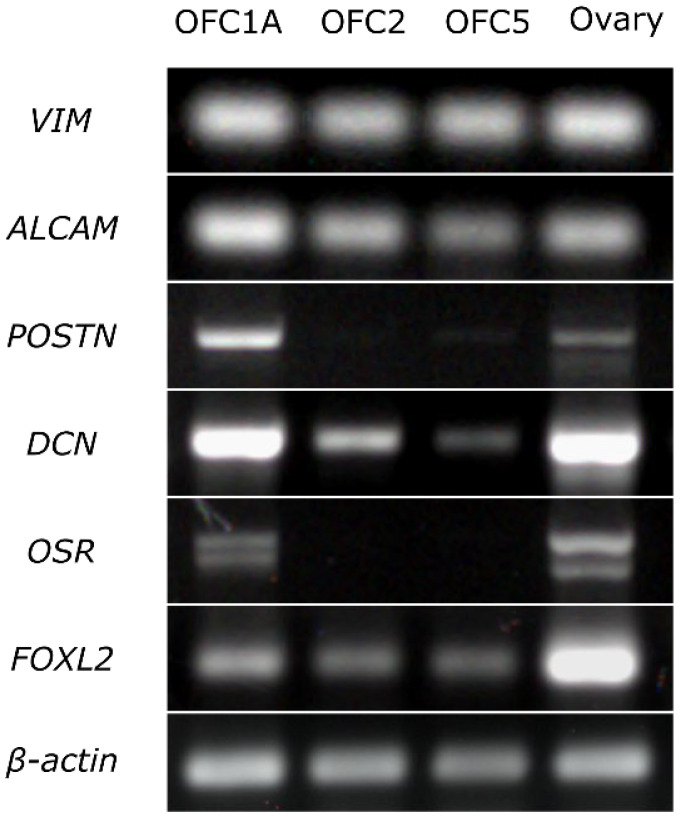
Expression of the markers specific to fibroblasts (*VIM*, *ALCAM*), interstitial cells (*POSTIN*, *DSN*) and granulosa cells (*OSR*, *FOXL2*) in OFC1A, OFC2 and OFC5 cell lines and in adult ovary detected by RT-PCR. All experimental and control samples were run on the same gel, except *β-actin* samples, which were run on a separate gel. Given that we detected faint *DCN* bands in RT-control, we considered the *DCN* band of the OFC5 sample as non-specific.

The results of RT-PCR indicate that all three cell lines expressed the fibroblast markers (*VIM*, *ALCAM*) and the marker of granulosa cells (*FOXL2*). The OFC1A line also expressed the markers of interstitial (stromal) cells (*POSTIN*, *DCN*) and granulosa cells (*OSR*) (Figure 3).

**Figure 4:**
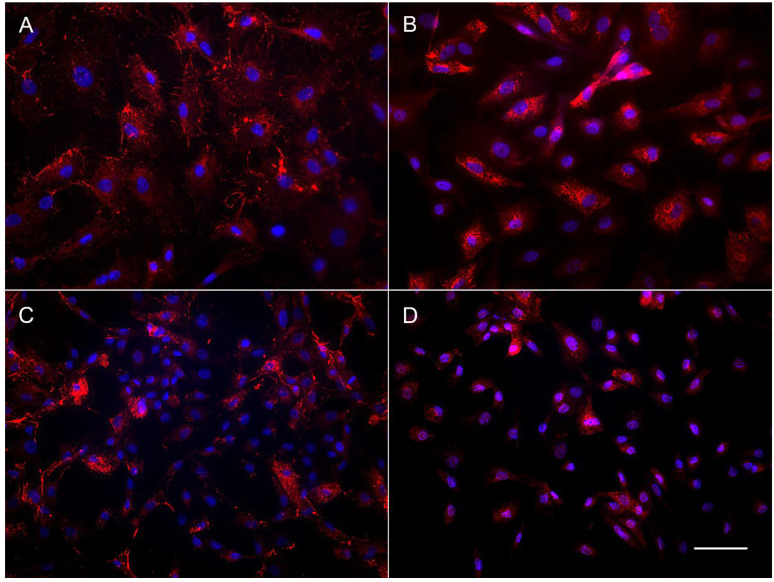
Immunolocalization of fibronectin (**A**,**C**) and collagen (**B**,**D**) in OFC5 (**A**,**B**) and OFC1A (**C**,**D**) cells. Bar—200 µm.

The results of immunocytochemical staining of the OFC cell cultures also revealed the difference in content between the subadult (OFC1A) and adult derived (OFC5) lines. The OFC5 line was uniformly stained by the antibodies for fibroblast-specific markers, fibronectin and collagen (Figure 4A,B). Meanwhile, the OFC1A cells varied in shape, size and morphology of the cells (Figure 4C,D), as well as in their staining pattern. In this cell culture, among the large cells brightly stained with antibodies for collagen and fibronectin, there are numerous small cells with a narrow rim of cytoplasm. This means that only some of the cells are fibroblasts.

Thus, all OFC lines contained the ovarian stroma cells. Meanwhile, OFC1A cell culture, which includes interstitial and granulosa cells together with fibroblasts, shows better growth characteristics than OFC2 and OFC5 cell lines, consisting mostly of fibroblasts. The variegated cell composition of the former line might be the cause of its shorter cell cycle and retention of proliferative activity for a longer time. The fibroblasts cell culture probably has less replicative potential and enters a senescence phase earlier than interstitial and granulosa cells. Alternatively, interstitial and granulosa cells might play the role of the feeder cells for the fibroblasts. Several studies on birds used the gonads’ stromal cells as the feeder cells for the embryonic and germ cell cultures [30,31].

The reasons for the variegated cell composition and higher proliferative activity of the OFC1A cell line remain unclear. The line has been derived from the ovary of a subadult female in September, which is beyond the breeding season, while the other three have been derived from adult females in May, during the breeding season. We do not know which factor (young age or the sexual rest period) was more important or if it was the mere chance that one of the lines was more productive than the other three.

### 3.3. Transfection and Electroporation with GFP Reporter Gene

We assessed the applicability of OFC1A cells for gene editing studies. Firstly, we checked the efficiency of their transduction with lentivirus containing GFP reporter in the presence of 10 and 50% of virus supernatant. These types of viral vectors are widely used for gene delivery to mammalian [32,33] and avian [34,35,36] cells. To produce transgenic birds, most of these studies injected the virus vectors into newly laid eggs. Due to features of avian development (chicken eggs already contain several tens of thousands of cells [37] and a large amount of yolk), the efficiency of that procedure in birds was lower compared to mammals.

In the last decade, avian primordial germ cells (PGCs) have become a promising tool for the production of transgenic birds. Several studies showed that they can be easily infected by the injection of a lentiviral vector directly into the blood vessels [38] or in cell cultures [30,39]. However, PGCs are difficult to obtain for non-laboratory birds.

We tried to enhance the efficiency of the transformation by using spinoculation, the process during which both the viral vector and the cells are centrifuged together to aid viral binding to the target cells, increasing the MOI for the different cell types [24,25]. Without spinoculation, the multiplicity of infection (MOI) for both OFC1A cells and skin fibroblasts of zebra finch was very low. Spinoculation increased the MOI in the OFC1A line, however, transduction efficacy remained relatively low. (Table 2).

Electroporation of the ovary-derived cells with the episomal vectors expressing GFP demonstrated much higher efficiency. The electroporated OFC1A cell culture contained about 50–70% of GFP-positive cells with a good survival rate (Figure S2; Table 3). The efficiency of the great tit fibroblast transformation under all protocols was lower. The zebra finch fibroblasts either died or did not express GFP at all.

Thus, we demonstrated that the established ovary-derived cell line could be efficiently used in gene engineering and gene editing studies. Although the efficiency of its transduction with lentivirus was very low, the electroporation provided a reasonably high efficiency of transformation.

## 4. Conclusions

We developed a simple method of primary somatic cell culture establishment from the ovaries and testes of Passerine birds. The ovary-derived cell cultures of the great tit were composed of fibroblasts mainly, but also contained interstitial and granulosa cells. They showed a high growth rate and high efficiency of genetic transformation after electroporation with episomal vectors expressing GFP. They are available on request and can be used in cytogenetic, genomic/gene engineering, and gene editing studies. The testis-derived cell cultures of the great tit and nine other Passerine species had lower proliferative potential than the ovary-derived ones. However, sufficient material was available from both cell cultures originating from the ovary and testis to produce high quality mitotic metaphase chromosomal preparations.

## Figures and Tables

**Table 1:** Comparison of the methods of bird chromosome preparation.

Source of Material	Advantages	Disadvantages	References
Bone marrow	Quick and simple protocol	Need to sacrifice birds; poor chromosome morphology	[26]
Feather pulp cell culture	No need to sacrifice birds; good chromosome morphology	Long procedure, fraught with failures; could not be applied to the small birds	[10,27]
Leukocyte cells culture	No need to sacrifice birds; good chromosome morphology	Long procedure, fraught with failures; could not be applied to the small birds	[28]
Skin fibroblast cells culture	No need to sacrifice birds; good chromosome morphology	Traumatic for small birds	[29]
Fetal fibroblasts cell culture	Relatively rapid procedure; good chromosome morphology	Difficult to obtain the material, which is available during the breeding season only	[11,12,13]
Gonads cell culture	Rapid procedure; good chromosome morphology; high and stable cell proliferation	Need to sacrifice birds	This paper

**Table 2:** The efficiency of the transformation of songbird cell lines with lentivirus containing GFP.

Cell Line	% of Lentivirus Containing Supernatant	Without Spinoculation			With Spinoculation		
		% of GFP-Positive Cells	10^5^ TU/mL	MOI	% of GFP-Positive Cells	10^5^ TU/mL	MOI
OFC1A	10	16	19.2	0.18	9.9	118.8	0.11
	50	9.8	23.5	0.11	37.0	88.8	0.46
Zebra finch fibroblasts	10	11.8	141.6	0.13	78.7	944.4	1.55
	50	50.6	121.4	0.71	42.6	102.24	0.56

**Table 3:** The efficiency of the electroporation of songbird cell lines with episomal vectors expressing GFP.

	Protocol	Pulse Voltage	Pulse Width	Pulse No	% of GFP-Positive Cells	Number of Cells Per Field-of-View
OFC1A	7	1200	30	1	79.0 ± 1.9	34.7 ± 1.4
	15	1300	20	2	70.7 ± 4.1	19.5 ± 0.9
	22	1400	10	3	53.5 ± 5.3	123.7 ± 5.9
Great tit fibroblasts	7	1200	30	1	54.5 ± 0.8	24.0 ± 0.7
	15	1300	20	2	66.3 ± 1.0	23.9 ± 0.4
	22	1600	10	3	59.1 ± 1.1	18.4 ± 0.5

## Data Availability

OFC1A cell culture is now available at the Collective Center of ICG SB RAS “Collection of Pluripotent Human and Mammalian Cell Cultures for Biological and Biomedical Research” upon request.

## Supplementary Materials

The following supporting information can be downloaded at: https://www.mdpi.com/article/10.3390/ani12131724/s1, Figure S1: The examples of metaphase chromosome plates prepared from the short term testicular cell cultures of Zebra finch (A), Gouldian finch (B), Common chaffinch (C), Brambling (D), European greenfinch (E), Domestic canary (F), European goldfinch (G) and Common linnet (H); Figure S2: The OFC1A cell culture electroporated by a plasmid with GFP; Table S1: Sources of the birds examined; Table S2: Primer sequences used for RT-PCR analysis.
