# Supplementary material for: Establishment of the Primary Avian Gonadal Somatic Cell Lines for Cytogenetic Studies
Source: Animals (Basel). 2022 Jul 4;12(13):1724. doi: 10.3390/ani12131724 (PMC9264790; doi:10.3390/ani12131724)
Supplement: Supplementary file 1 [file animals-12-01724-s001.zip › animals-1785384-supplementary.pdf]

Supplementary Material

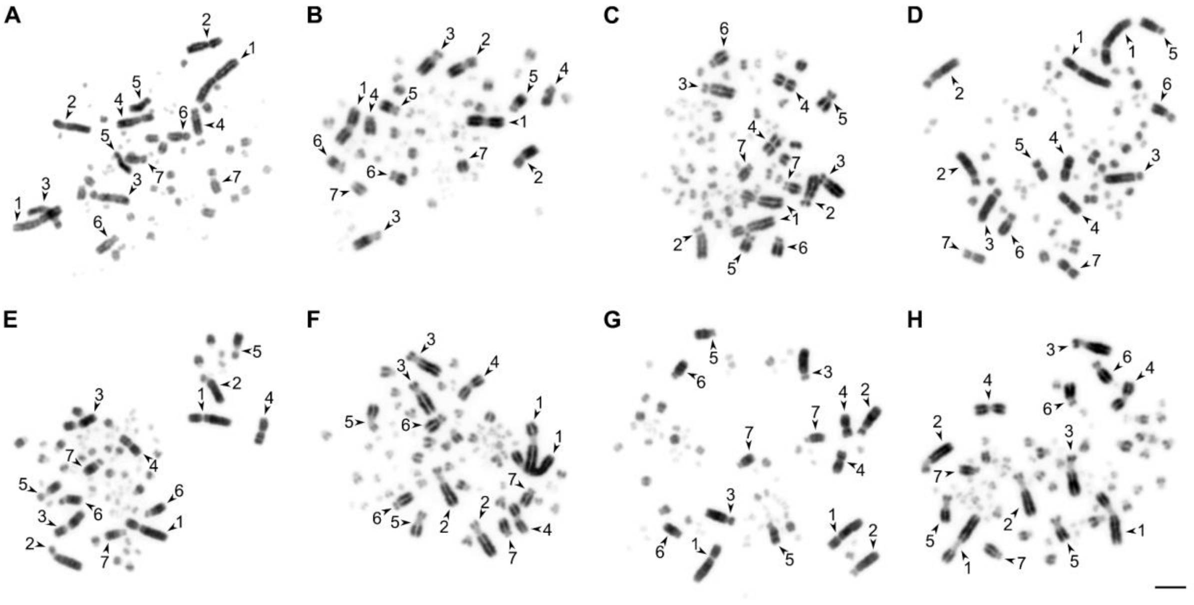

Figure S1. The examples of metaphase chromosome plates prepared from the short term testicular cell cultures of zebra finch (A), Gouldian finch (B), common chaffinch (C), brambling (D), European greenfinch (E), domestic canary (F), European goldfinch (G) and common linnet (H). The arrowheads indicate the seven largest macrochromosomes. Bar – 5 µm.

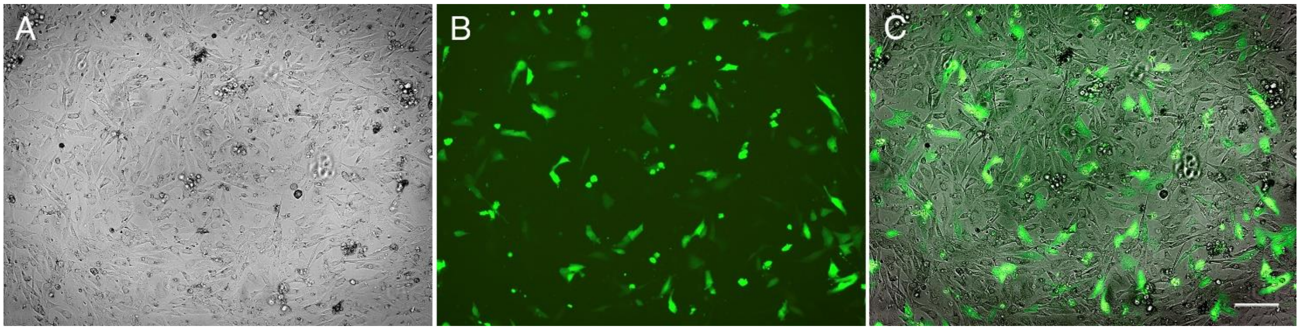

Figure S2. The OFC1A cell culture electroporated by plasmid with GFP. A - phase contrast image, B - GFP, C - merged image. Bar - 100µm.

Table S1. Sources of the birds examined

Scientific name	Common name	No of specimens	Source
Parus major	Great tit	4 females, 1 male	Trapped in Novosibirsk
Bathilda ruficauda	Starfinch	1 male	Purchased from a commercial breeder
Taeniopygia guttata	Zebra finch	1 male	Purchased from a commercial breeder
Chloebia gouldiae	Gouldian finch	1 male	Purchased from a commercial breeder
Fringilla coelebs	Common chaffinch	1 male	Trapped in Novosibirsk
Fringilla montifringilla	Brambling	1 male	Trapped in Novosibirsk
Chloris chloris	European greenfinch	1 male	Trapped in Novosibirsk
Serinus canaria forma domestica	Domestic canary	1 male	Purchased from a commercial breeder
Carduelis carduelis	European goldfinch	1 male	Trapped in Novosibirsk
Linaria cannabina	Common linnet	1 male	Trapped in Novosibirsk

Table S2. Primer sequences used for RT-PCR Analysis

Gene	Sequence (5′–3′)		Product size(bp)
	Sense	Antisense	
VIM	TAGTTGGCGAAGCGGTCATT	GACTCGGTGGACTTCACCTT	107
ALCAM	ACCAGACTACAAAGACAGGTTGA	GACTATCGTCGGCTCTTCGG	136
POSTN	CTCCAGACCCTTTGCCAGTT	CTCCCCTCGGAAGTCTCTCA	918
DCN	AGGTGCCAAAAGACCTTCCC	TGCCAAGTTGGTGAGTCCAG	524
OSR1	GGAGCAGTGGGCTCCAATTT	CCGCTAGTGTTCTGGACTGG	838
FOXL2	TGGGAGCATGACAGCAAACA	TACACTGACACTACACGCGG	234
β-actin	CTCGAAATCCAGGGCGACAT	CTCATGCCATCCTCCGTCTG	158
